# First report of the nematode *Cruzia tentaculata* using molluscs as natural intermediate hosts, based on morphology and genetic markers

**DOI:** 10.1016/j.ijppaw.2021.02.013

**Published:** 2021-02-23

**Authors:** Jucicleide Ramos-de-Souza, Arnaldo Maldonado-Jr, Roberto V. Vilela, Beatriz E. Andrade-Silva, Helene S. Barbosa, Suzete R. Gomes, Silvana C. Thiengo

**Affiliations:** a Programa de Pós-Graduação em Biologia Parasitária, Instituto Oswaldo Cruz / Fundação Oswaldo Cruz, Av. Brasil 4365, Rio de Janeiro, RJ, 21040-360, Brazil; b Laboratório de Biologia e Parasitologia de Mamíferos Silvestres Reservatórios, Instituto Oswaldo Cruz / Fundação Oswaldo Cruz, Av. Brasil 4365, Rio de Janeiro, RJ, 21040-360, Brazil; c Laboratório de Referência Nacional para Esquistossomose - Malacologia, Instituto Oswaldo Cruz / Fundação Oswaldo Cruz, Av. Brasil 4365, Rio de Janeiro, RJ, 21040-360, Brazil; d Laboratório de Biologia Estrutural, Instituto Oswaldo Cruz / Fundação Oswaldo Cruz, Av. Brasil, 4365 Manguinhos, Rio de Janeiro, RJ, 21045-900, Brazil

**Keywords:** MT-CO1, 18S rRNA, *Strongyluris* sp., *Achatina fulica*, *Latipes erinaceus*, *Thaumastus taunaisii*

## Abstract

The life cycles of many parasitic nematodes include terrestrial gastropods as intermediate hosts. Over the past few decades, a number of cases of parasitism between molluscs and medically-important nematodes have been reported in Brazil, in particular, those involving the invasive giant African gastropod, *Achatina fulica*, and zoonoses caused by the nematodes *Angiostrongylus cantonensis* and *Angiostrongylus costaricensis*, the etiological agents of neuroangiostrongyliasis and abdominal angiostrongyliasis, respectively. In the present study, larvae found infecting *A. fulica*, *Latipes erinaceus*, and *Thaumastus taunaisii*, from two localities in the Brazilian state of Rio de Janeiro were characterized using light and scanning electron microscopy, and sequences of the 18S rRNA and MT-CO1 genes. Genetic markers allowed to identify the larvae collected in the present study as *Cruzia tentaculata,* whose adults parasitize didelphid marsupials in the Americas. These findings indicate that both native and non-native gastropods may act as intermediate hosts and represent a previously unnoticed heteroxenous life cycle of *C. tentaculata*.

## Highlights

• First report of *Cruzia tentaculata* as heteroxenous life cycle involving *A. fulica*.
• Integrative taxonomy of *C. tentaculata* larvae by morphology and MT-CO1 and 18S rRNA.
• *C. tentaculata* in both opossum and terrestrial molluscs.
• Infected molluscs with *C. tentaculata* have been found in urban and preserved areas.
• *Strongyluris*-Like reported in previous studies infecting *A. fulica* may represent *C. tentaculata.*

## Graphical abstract

**Figure:**
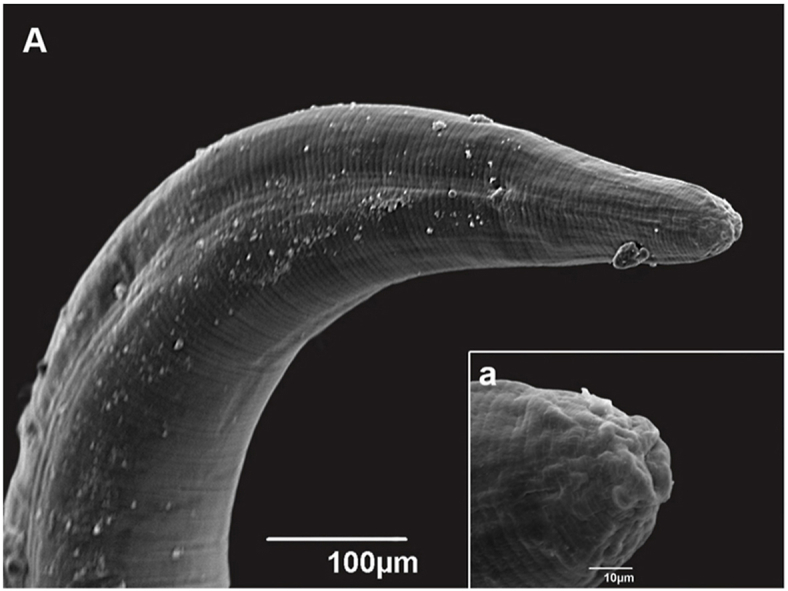

## 1 Introduction

Molluscs can act as vectors in the transmission of parasitic worms of pets, livestock and wildlife, thus contributing to the spread of zoonoses through their dispersal capacity. The giant African land snail, *Achatina fulica* Bowdich, 1822, an invasive species from Africa, which is currently found in Asia and Oceania, recently has been spreading throughout South America and is also present in Florida, USA (Fontanilla et al., 2014). Associated with the spread of *A. fulica*, the zoonotic nematode *Angiostrongylus cantonensis* (Chen, 1935) has been confirmed as one causative agent of parasitic eosinophilic meningitis in human populations of the Americas (Morassutti et al., 2014; Ramos-de-Souza et al., 2018; Valente et al., 2018).

In South and Central America, several nematodes have been detected infecting *A. fulica*, i.e., *Aelurostrongylus abstrusus* (Railliet, 1898), parasite that infects the lungs of felines and *Strongyluris* sp., parasite of lizards (Thiengo, 1995; Thiengo et al., 2008; Oliveira et al., 2010; Pereira et al., 2017; Ramos-de-Souza et al., 2018). This snail is also considered a potential host for *Angiostrongylus costaricensis* Morera & Céspedes 1971 (Carvalho et al., 2003). During the past few years, we have collected a large number of *A. fulica* individuals naturally infected by larvae resembling *Strongyluris* spp. and *A. cantonensis*. This drew our attention to the potential susceptibility of *A. fulica* to nematodes present in areas it has recently invaded and its capacity for their dissemination over a large geographical scale within a short period of time which is strongly influenced by human activities. Since *A. fulica* has a high reproductive rate, potential for dispersal, and compatibility with helminths of humans, livestock, and pets (Thiengo et al., 2007, 2008), it may play an important role in disseminating parasitic worms of indigenous fauna.

Recent parasitological surveys of molluscs in the state of Rio de Janeiro have recovered several different forms of nematode larvae, including some belonging to unidentified taxa, highlighting the possibility of a role for *A. fulica* in infection of the region's wildlife. The present study detected and described unidentified nematode larvae recovered from the invasive *A. fulica* and from two aboriginal gastropods – *Thaumastus taunaisii* (Férussac, 1822) and *Latipes erinaceus* (Colosi, 1922) – in the state of Rio de Janeiro, Brazil. The larvae were identified based on morphology and molecular analysis of nuclear 18S rRNA and mitocondrial MT-CO1 genes.

## 2 Materials and methods

Nematode larvae of a single morphotype were recovered from three mollusc species collected from three sites in the state of Rio de Janeiro, Brazil. Individuals of two of the molluscs (*A. fulica* and *T. taunaisii*) were collected in the municipality of Rio de Janeiro. Invasive snails *A. fulica* (n = 7) were collected at the Fiocruz Manguinhos Campus (22°52′31.2″S, 43°14′51.4W), whereas native snails *T. taunaisii* (n = 2) were obtained from the Fiocruz Atlantic Forest Campus (CFMA: 22°55′27.5″S, 43°26′27.0″W), adjoining the Pedra Branca State Park (*Parque Estadual da Pedra Branca* - *PEPB*). A single individual of the autochthonous slug *Latipes erinaceus* was collected in the municipality of Paraty (23°13′01.8″S, 44°43′22.5″W). The molluscs were collected between November 2017 and April 2019, and in all cases (except for *L. erinaceus* and one *A. fulica* individual) the parasitological analysis was based on artificial digestion of the molluscs (Graeff-Teixeira and Morera, 1995). In addition, we also added to this study, adult worms identified as *Cruzia tentaculata*, recovered from opossum *Didelphis aurita* from Fiocruz Mata Atlântica Campus.

### 2.1 Morphological analyses

Larvae recovered from each mollusc were fixed in AFA (2% glacial acetic acid, 3% formaldehyde, 95% ethanol) for morphological analyses (light microscopy – LM, and Scanning Electron Microscopy – SEM). The AFA-fixed specimens were clarified in lactophenol (50% lactic acid, 25% phenol, 25% distilled water) for description of morphological structures: body width, nerve ring, muscular and glandular esophagus, esophageal bulb, pre-bulb, excretory pore, and tail. The morphological structures were classified following Travassos (1917; 1922), and the specimens were identified using taxonomic keys (Anderson et al., 2009; Adnet et al., 2009; Gibbons 2010).

For SEM, the nematode larvae collected from *A. fulica* (3 larvae), *L. erinaceus* (2), and *T. taunaisii* (1) were processed according to Mafra and Lanfredi (1998). The samples were analyzed in a JEOL JSM-6390 microscope (Tokyo, Japan) at the Rudolf Barth Electron Microscopy Platform of the Oswaldo Cruz Institute, in Rio de Janeiro.

### 2.2 Molecular analyses

Three larvae recovered from *A. fulica*, two from *L. erinaceus*, and two from *T. taunaisii* were transferred to 70% ethanol for DNA extraction and molecular analyses. The samples were washed individually in distilled water for 24 h. The DNA was then extracted using a QIAamp DNA Mini kit (QIAGEN, Hilden, Germany), following the manufacturer's protocol.

The partial nuclear small subunit ribosomal RNA gene (18S rRNA) sequence was amplified by conventional Polymerase Chain Reaction (PCR) using the primer pair Physa_F and Physa_R (Gomes et al., 2015). The PCR reactions had 12.5 μL of PCR Master Mix (PROMEGA, Madison, USA), 0.5 μL of each primer (10 μM each), 3.0 μL of the genomic DNA, and ultrapure water to complete a total reaction volume of 25 μL. The thermal cycling conditions followed Gomes et al. (2015).

The barcode region of the mitochondrial cytochrome *c* oxidase subunit I gene (MT-CO1) was amplified using the primer cocktail of Prosser et al. (2013). The PCR reactions had 12.5 μL of PCR Master Mix (PROMEGA, Madison, USA), 0.5 μL of each primer cocktail (10 μM of a three-forward-primers mix and 10 μM of a three-reverse-primers mix), 3.0 μL of genomic DNA, and ultrapure water to complete a total reaction volume of 25 μL. The thermal cycling conditions followed Prosser et al. (2013).

After 1.5% agarose gel electrophoresis and visualization on UV transilluminator, successfully amplified samples were purified using the Illustra GFX PCR DNA and Gel Band Purification kit (GE Healthcare Little Chalfont, Bucks, UK) following the manufacturer's protocol. Cycle-sequencing reactions were conducted using the BigDye Terminator v3.1 Cycle Sequencing kit (Applied Biosystems, Carlsbad, California, USA), reactions were run individually for each primer for better accuracy. The samples were sequenced in an ABI 3730 DNA Analyzer (Applied Biosystems) at the DNA Sequencing Platform of the Oswaldo Cruz Institute, PDTIS/FIOCRUZ, subunit RPT01A – DNA Sequencing.

We searched GenBank (www.ncbi.nlm.nih.gov/genbank/) for similar sequences using BLAST (Basic Local Alignment Search Tool), firstly with 18S rRNA sequences and subsequently with MT-CO1 sequences, based on observations of the first results. Our sequences were assembled into contigs and edited using the Geneious R9 software package (Kearse et al., 2012). From the BLAST results, for phylogenetic analyses, we added other nematode species sequences from GenBank, representing the superfamily Cosmocercoidea, as outgroup we included sequences representing the superfamily Heterakoidea, based on this superfamily phylogenetic proximity to Cosmocercoidea (Supplementary file S1).

The 18S rRNA sequences were aligned using the SINA Aligner v1.2.11 (Pruesse et al., 2012), while the MT-CO1 sequences were aligned using the Translator X server (Abascal et al., 2010). Each resulting matrix was trimmed to eliminate poorly-aligned extremities, and converted to different formats using Mesquite version 3.51 (Maddison and Maddison, 2018). Bayesian Inference (BI) analyses were run in MrBayes version 3.2.6 (Ronquist et al., 2012) with GTR + I + G model command blocks added to the matrices using Mesquite version 3.51 (Maddison and Maddison, 2018). MrBayes analyses were run in the CIPRES Science Gateway V. 3.3 (Miller et al., 2010).

## 3 Results

### 3.1 Morphological analyses by light and scanning electron microscopy

Nine of ten molluscs collected in the present study were infected by whitish robust non-identified larvae. The number of larvae recovered per individual varied considerably in *A. fulica*, ranging from three to 70, whereas two and five larvae were obtained from the two *T. taunaisii* individuals, and eight larvae were collected from *L. erinaceus*.

**Fig. 1:**
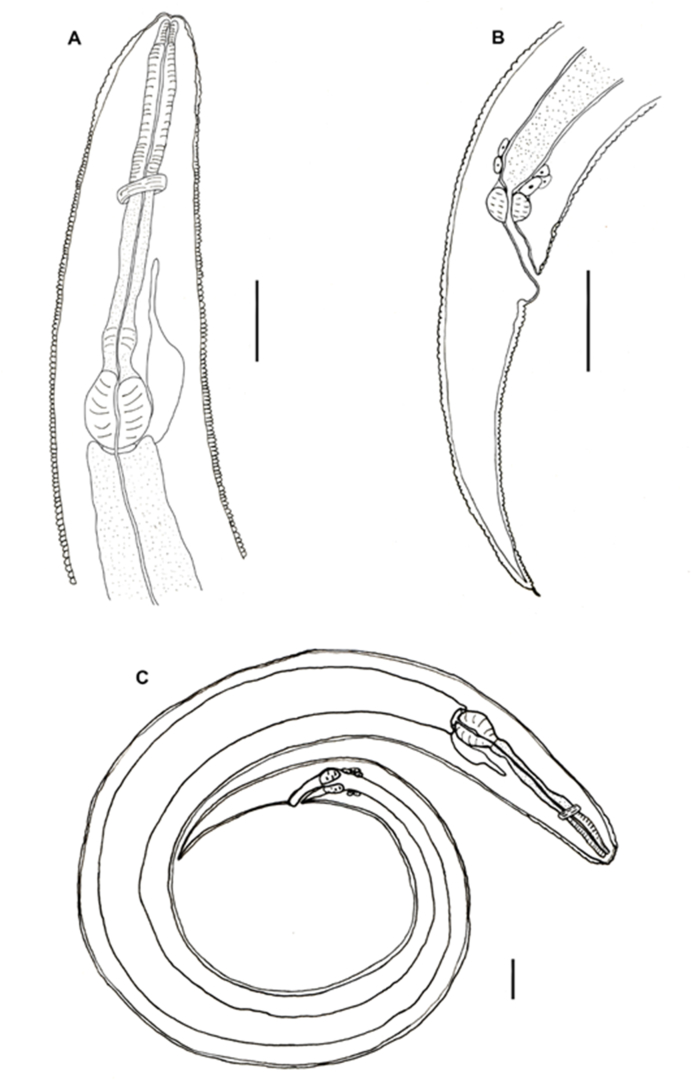
Line drawings of *Cruzia tentaculata* recovered from the molluscs in the present study, based on light microscopy. A) side view of the anterior region of a nematode recovered from *Achatina fulica*; B) side view of the posterior region of a nematode from *A. fulica*; C) Lateral view of a whole specimen recovered from *Latipes erinaceus*. Scale bar = 100 μm.

**Fig. 2:**
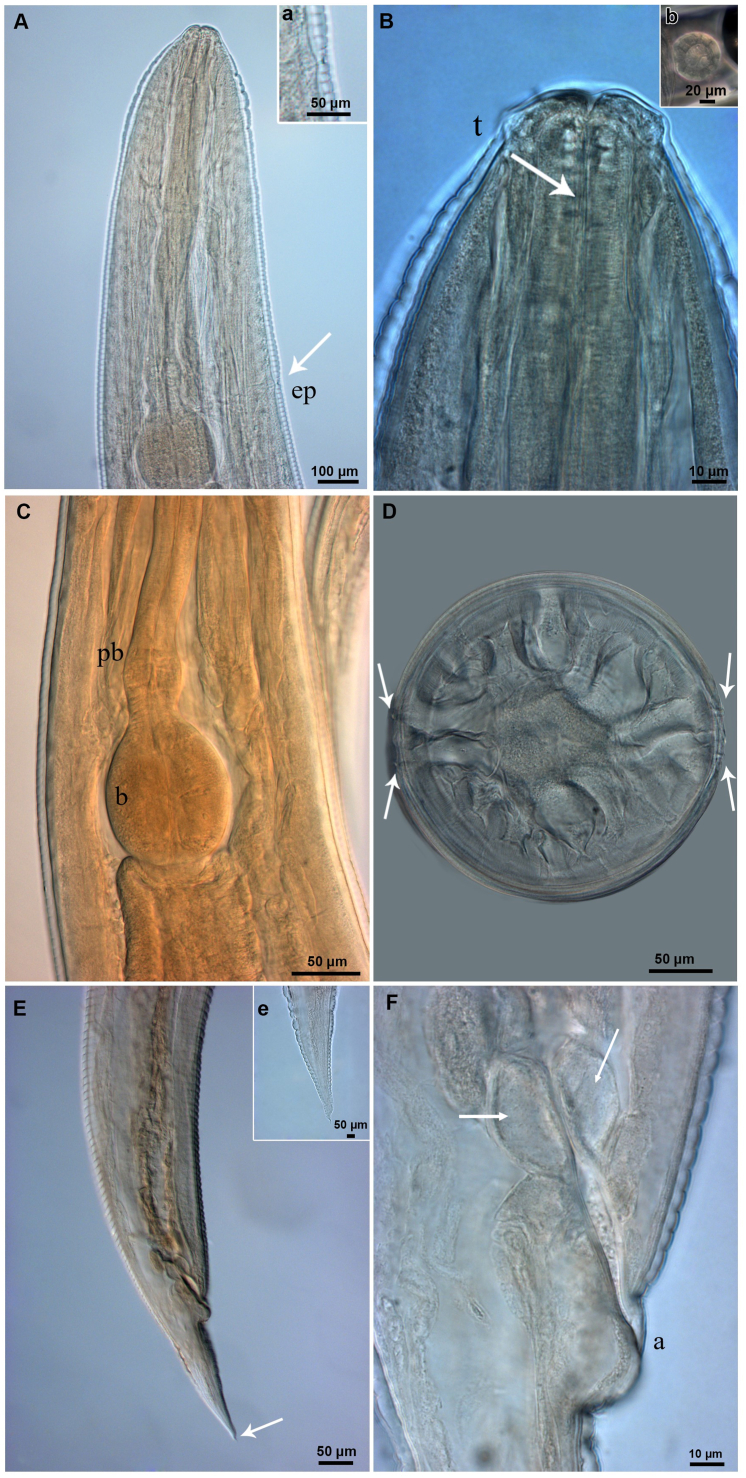
Photomicrographs of the *Cruzia tentaculata* larvae recovered from *A. fulica*; A) Anterior extremity, showing the excretory pore (ep), bulb, and pre-bulbar dilatation; (a) details of the excretory pore in lateral view; B) Cephalic extremity showing the labial papillae and the teeth (t), apical view; b) Trilabial mouth, apical view; C) Pre-bulbar dilatation (pb) and well developed bulb; D) Lateral lines (arrows), transversal section; E) Posterior extremity, lateral view; (e) detail of the extremity of the tail; F) anus (a), with prominent opening lateral view and a pair of anal glands (arrow).

**Fig. 3:**
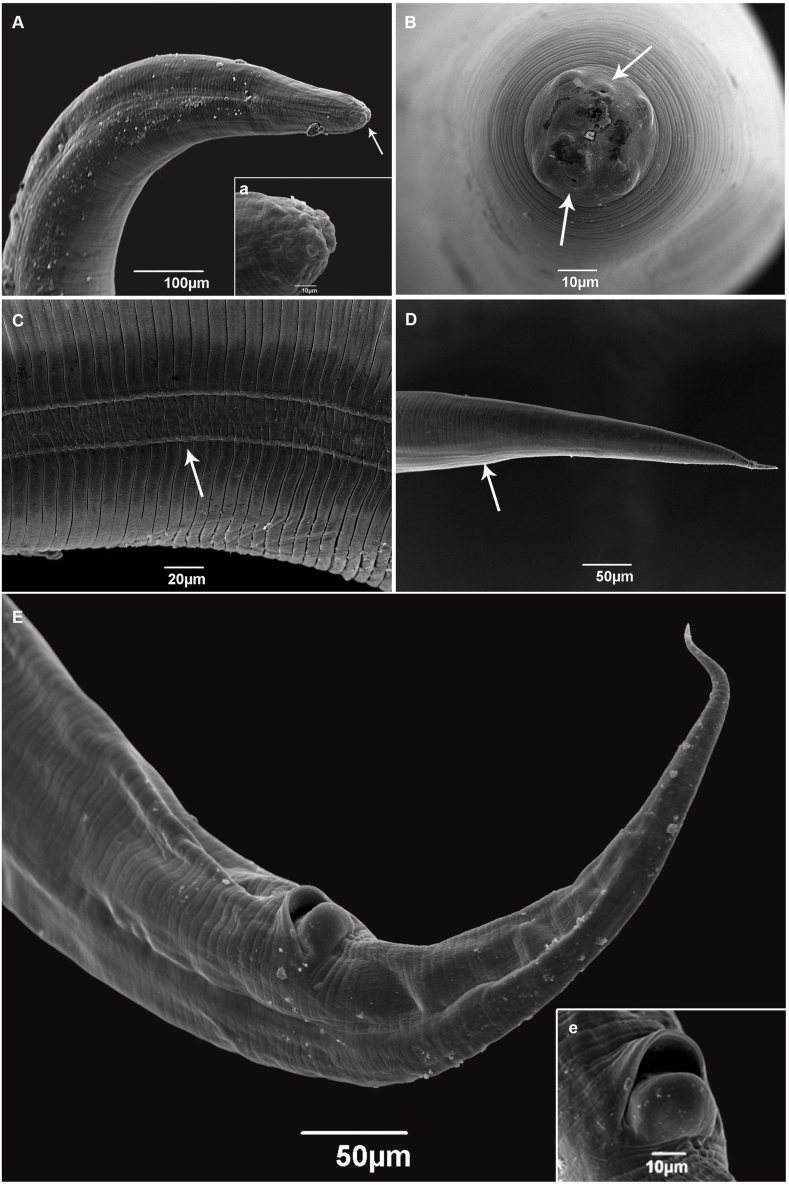
Scanning Electron Microscope images of a *Cruzia tentaculata* larvae recovered from *Achatina fulica*: A) Anterior extremity and detail of the apical view showing the oral opening; B) Cephalic extremity with labial papillae and phasmids (arrows), apical view; C) Lateral view showing the lateral line (arrow); D) posterior region, lateral view showing the lateral line (arrow); E) posterior region, ventral view, and (e) detail of the anus.

All larvae examined had an elongated body, with a lanceolate tail (Fig. 1). Under light microscopy, the larvae exhibited a long esophagus, divided into anterior (muscular) and posterior (glandular) parts, followed by a discrete pre-bulbar dilation, a well-developed bulb (Fig. 1A and 2 C), and a discrete intestinal diverticulum, projecting anteriorly to the level of the pre-bulbar dilation (Fig. 1A); an excretory pore located near the bulb (Fig. 2A); buccal cavity lined with at least one row of hooks in the lateral view (Fig. 2B); a double lateral line running along the side of the body (Figs. 2D, 3C and 3D); posterior region conical-shaped with a sharply pointed tail (Fig. 2, Fig. 3E); anal opening with prominent anterior edge situated near the end of the body, preceded by a pair of anal glands (Fig. 2, Fig. 3E). The structures visible in the SEM included the lateral line, poorly defined lips, anus with prominent border, and pointed tail (Fig. 3).

The larval specimens – five from *A. fulica*, and two each from *L. erinaceus* and *T. taunaisii* were deposited in the Helminthological Collection of the Instituto Oswaldo Cruz, under catalog numbers CHIOC 38721–38723. Adult worms (n = 2), identified as *Cruzia tentaculata*, recovered from opossum *Didelphis aurita*, were also deposited under the collection number CHIOC 38782.

### 3.2 Molecular analyses

The partial sequencing of the 18S rRNA resulted in two good quality chromatograms (forward and reverse) of over 800 base pairs (bp) for each sample. As the larvae obtained from the three mollusc hosts, together with the adult *C. tentaculata* recovered from *D. aurita*, all shared the same 18S rRNA gene sequence, only one sequence was included in the subsequent analyses.

The partial sequencing of the MT-CO1 produced six sequences of nearly 700 bp for each sample. Our sequences were deposited in GenBank under accession numbers MN873564, MN873565, MN873566, and MN873570 for the 18S rRNA, and MN842776, MN842777, and MN842778 for the MT-CO1 (Supplementary file S1).

The 18S rRNA sequence of the larvae recovered from the molluscs, and the sequence of the adult *C. tentaculata*, recovered from *D. aurita* formed a well-supported monophyletic group with the GenBank sequence of *Cruzia americana* (BPP = 1.00) (Supplementary file S2). The MT-CO1 sequences of the larvae and the adult *C. tentaculata* formed together a well-supported monophyletic group (BPP = 1.00) (Supplementary file S3).

In the MT-CO1 analyses, two of three larvae yielded good quality sequences, from which, two haplotypes were obtained. A third haplotype, of the adult *C. tentaculata* from *D. aurita*, was distinct from that of either larval haplotypes, that nevertheless formed a moderately-supported monophyletic group with the sequences of larvae (BPP = 0.60), sister to the adult haplotype.

## 4 Discussion

The morphological and genetic analyses confirmed the identification of the larvae recovered from the cysts found in the pallial cavity of *Achatina fulica* and *Thaumastus taunaisii*, and the body cavity of *Latipes erinaceus* as *Cruzia tentaculata*, a known parasite of the cecum of Neotropical didelphid marsupials (Anderson et al., 2009).

### 4.1 Taxonomy and distribution

In total, 13 *Cruzia* species are currently recognized, including parasites of amphibians, reptiles, marsupials and xenarthrans (Anderson et al., 2009; Adnet et al., 2009; Li, 2019; Vieira et al., 2020). Among marsupial hosts, three species are known *Cruzia cameroni* Wolfgang, 1951; *C. americana* and *C. tentaculata* (Li, 2019). *Cruzia tentaculata* was described originally as *Ascaris tentaculata* Rudolphi, 1819 (Travassos, 1917, 1922) and assigned to the family Ascarididae, was subsequently placed by Travassos (1917) in his new genus *Cruzia* in a new family Cruzidae, with a single species, *Cruzia tentaculata*. Subsequently, *C. tentaculata* was placed within the family Kathlaniidae (Travassos, 1922; Anderson et al., 2009). *Cruzia americana,* a parasite of the cecum and large intestine of the opossum *Didelphis virginiana* in the United States, may cause severe pathology, or even death, at high infestation rates (Nichelason et al., 2008; Anderson et al., 2009). *Cruzia tentaculata* and *C. americana* both occur in didelphid marsupials, although there are records of armadillo (Dasypodidae) as hosts, in both South and North America, in particular in Brazil, Colombia, Paraguay, and Mexico (Travassos, 1922; Adnet et al., 2009; Li, 2019). In Brazil, there are reports of *C. tentaculata* parasitizing opossums in both the Amazon and the Atlantic Forest, including the state of Rio de Janeiro (Travassos, 1922; Adnet et al., 2009). Until now, however, nothing was known of an intermediate host.

The generalist dietary habits of the didelphid opossums, their tolerance of anthropogenic environments, and the presence of *A. fulica*, an invasive mollusc, in the same habitats, may favor the life cycle of *C. tentaculata*. This is probably reinforced by the fact that *A. fulica* is widely distributed in Brazil and normally occurs in dense populations due to its high reproductive potential and generalist habits (Thiengo et al., 2013). Given this, *A. fulica* is presumably a novel, and epidemiologically important species that may transmit this parasite to wild mammals, forming a link between the parasite and its definitive host in urban and peri-urban areas. The other mollusc species analyzed in the present study, *L. erinaceus* and *T. taunaisii*, are autochthonous to Brazil and may act as natural intermediate hosts of *C. tentaculata.* Also it is possible that other native species of gastropods participate of this life cycle.

### 4.2 Biological features

The nematode larvae were invariably observed encysted in the pallial or body cavity of the molluscs, with up to 70 larvae being found in a single individual. The most frequent form of the larvae, probably the L_3_ stage, was found in all mollusc species. Oliveira and Santos (2018) concluded tentatively that the larvae recovered from *A. fulica* may have hatched after the ingestion of the eggs by the mollusc, with these larvae then becoming encysted in the pallial cavity, where they encountered suitable glycogen storage that allowed them to develop to the L3 stage, thus indicating that these molluscs are intermediate hosts.

Valente et al. (2016) suggested that the presence of these larvae in the molluscs may represent an abortive cycle, in which they failed to complete their stage of life cycle in the molluscs. In the present study, however, the life cycle of *C. tentaculata* apparently was not interrupted within the mollusc, given that the encysted larvae were still alive. It seems possible that the reserves of glycogen in the mollusc tissues may have supported the parasitism (Oliveira and Santos, 2018).

Molluscs are also a part of the diet of opossums (Franco-Acuña et al., 2009; Li, 2019), which are the definitive hosts of *C. tentaculata* (Travassos, 1922; Adnet et al., 2009), so it is possible that the life cycle of the parasite includes the infection of gastropods when these invertebrates ingest opossum feces containing nematode eggs. The biological compatibility of different host molluscs further supports their potential role as intermediate hosts for this nematode.

### 4.3 Morphological features

The larvae recovered from the molluscs in the present study were morphologically similar to the *Strongyluris* sp. larvae reported previously (Thiengo, 1995, Oliveira et al., 2010; Valente et al., 2016). However, the morphological comparison of our samples with the original descriptions of adult *Cruzia* sp. and subsequent papers, revealed similar structures in both the adult and the larvae (Thiengo, 1995; Travassos, 1917, 1922; Anderson et al., 2009). Given these similarities, the specimens were identified as *Cruzia* sp., based on the presence of papillae, trilabial mouth, long tail, the position of the excretory pore, presence of a pre-bulbar dilatation, buccal cavity with pharyngeal teeth, lateral line on the body, a discrete intestinal diverticulum projecting anteriorly, and the anal protuberance and mainly the buccal cavity with longitudinal row of cuticular lamellae.

### 4.4 Molecular features

Our molecular 18S rRNA analyses suggested a close relationship between the larvae collected in the present study and *Cruzia americana*. Given the absence of *C. americana* MT-CO1 sequences in GenBank, we included the MT-CO1 sequence of an adult *C. tentaculata* recovered from *Didelphis aurita*. This confirmed that our samples had similar haplotypes, thus supporting that all samples represented the same species, *C. tentaculata*. Since the larvae analyzed in the present study were obtained from different gastropod species (both native and non-native), and distinct habitats, *i.e.*, well-preserved Atlantic Forest and anthropogenic environments, it seems likely that *Cruzia tentaculata* has a low degree of specificity in terms of either its intermediate host or the environments in which it occurs.

## 5 Conclusions

Our study highlights the urgent need for a comprehensive reassessment of the helminth fauna of terrestrial gastropods. It is the first to provide molecular and morphological evidence on the occurrence of *Cruzia tentaculata* larvae in terrestrial molluscs, including both native and invasive species, further contributing with DNA sequences of adult *C. tentaculata* from an opossum. Prior to the present study, the participation of molluscs in the life cycle of *C. tentaculata* had been entirely overlooked, and this is the first record of the role of terrestrial molluscs as intermediate hosts in the life cycle of *C. tentaculata*. These findings also indicate that the previous studies that have identified *Strongyluris* sp. infecting molluscs, based only on the larval morphology, may in fact have misidentified *C. tentaculata*. It may thus even be possible that *Strongyluris* does not infect molluscs at all, and further research, based on molecular analyses of such larvae, would be required to confirm this.

## Availability of data and materials

All data generated or analyzed during this study are included in this published article and its additional files.

## Ethics approval and consent to participate

Not applicable.

## Consent for publication

Not applicable.

## Competing interests

The authors declare that they have no competing interests.

## Funding

This study was funded by VPEIC/FIOCRUZ.

## Author contributions

JRS, SCT and AMJ conceived, designed, and supervised the study. JRS, RVV, BEAS, HSB and SRG conducted the study. JRS analyzed the data and wrote the manuscript, which was further revised and edited by AMJ, RVV, and SCT. All the authors read and approved the final manuscript.

## Declaration of competing interest

All the authors declare that they have no competing interests.
