# Supplementary material for: First report of the nematode Cruzia tentaculata using molluscs as natural intermediate hosts, based on morphology and genetic markers
Source: Int J Parasitol Parasites Wildl. 2021 Feb 23;15:105–11. doi: 10.1016/j.ijppaw.2021.02.013 (PMC8102712; doi:10.1016/j.ijppaw.2021.02.013)
Supplement: Multimedia component 2 [file mmc2.docx]

**Supplementary file S1.** Nematode species included in the phylogenetic analyses of the present study, their respective taxonomic affinities, hosts, GenBank accession numbers for the 18S rRNA and MT-CO1 sequences, and references.

**Supplementary file S2.** Bayesian Inference phylogenetic tree of partial 18S rRNA gene sequences of representative species of Cosmocercoidea, with the Heterakoidea species as outgroup. Numbers at the nodes are Bayesian posterior probabilities (BPPs), based on 50% majority-rule consensus of the sampled trees after 25% burn-in. The monophyletic group formed by *Cruzia americana* and the samples from the present study (*C. tentaculata*) is highlighted in red.

**Supplementary file S3.** Bayesian Inference phylogenetic tree of partial MT-CO1 gene sequences of representative species of Cosmocercoidea, with Heterakoidea species as outgroup. Numbers at the nodes are Bayesian posterior probabilities (BPPs), based on 50% majority-rule consensus of the sampled trees after 25% burn-in. The monophyletic group formed by *Cruzia tentaculata* larvae recovered from *Achatina fulica* and the adult worm from *Didelphis aurita* (present study) is highlighted in red.
